# Methicillin-Resistant *Staphylococcus aureus* ST398 from Human Patients, Upper Austria

**DOI:** 10.3201/eid1505.080326

**Published:** 2009-05

**Authors:** Karina Krziwanek, Sigrid Metz-Gercek, Helmut Mittermayer

**Affiliations:** Austrian National Reference Centre for Nosocomial Infections and Antibiotic Resistance, Linz, Austria

**Keywords:** Antimicrobial resistance, zoonoses, staphylococci, Staphylococcus aureus, MRSA, Austria, ST398, pigs, animals, dispatch

## Abstract

Methicillin-resistant *Staphylococcus aureus* (MRSA) clonal type ST398 is usually associated with animals. We examined 1,098 confirmed MRSA samples from human patients and found that 21 were MRSA ST398. Most (16) patients were farmers. Increasing prevalence from 1.3% (2006) to 2.5% (2008) shows emergence of MRSA ST398 in humans in Austria.

In the past few years, interest has focused on the emergence of methicillin-resistant *Staphylococcus aureus* (MRSA) in animals and the potential for cross-transmission between humans and animals (*1*). MRSA isolates that are strongly associated with pigs or contact with pigs show at least 2 similarities: they are not typeable by pulsed-field gel electrophoresis (PFGE) because their DNA cannot be digested by the restriction enzyme *Sma*I (*2*), and most belong to the MRSA clonal lineage sequence type (ST) 398 (*1*).

## The Study

From January 2006 through May 2008, a total of 21 laboratories and/or hospitals in Upper Austria (project MRSA-Registry Upper Austria) sent us 1,210 suspected MRSA primary isolates consecutively collected from human patients. For quality control, all isolates were cultured and investigated for *mecA/femA* by PCR at the Austrian National Reference Centre (*3*). Of the 1,210 isolates, 1,098 (90.7%) were confirmed to be MRSA; the other 112 (9.3%) were either methicillin-sensitive *S. aureus* or were not *S. aureus* and therefore were excluded from the MRSA registry.

Most molecular biological investigations (DNA isolation, detection of the Panton-Valentine leukocidin [PVL] genes *lukS-lukF*, PFGE, *spa* typing) were performed as described (*3*). Determination of staphylococcal cassette chromosome *mec* (SCC*mec*) subtypes was performed by PCR according to Boye et al. (*4*). Multilocus sequence typing (MLST) PCR was performed according to Enright et al. (*5*). Sequence reactions were conducted by using BigDye fluorescent terminators (Applied Biosystems, Foster City, CA, USA). Sequencing was performed on the ABI PRISM 310 Genetic Analyzer (Applied Biosystems). Sequence types were assigned according to the *S. aureus* MLST database with use of the typing software at www.mlst.net (*6*).

Etest showed resistance patterns of the MRSA ST398 isolates to the following antimicrobial agents: ciprofloxacin, clindamycin, erythromycin, fosfomycin, fusidic acid, gentamicin, moxifloxacin, mupirocin, rifampin, and vancomycin. Resistance to doxycycline was determined by using the disk diffusion test. Data were interpreted according to the guidelines of the Clinical and Laboratory Standards Institute antimicrobial susceptibility testing standards, where available.

Demographic information and data from patient histories were systematically collected by using standardized questionnaires and were interpreted by physicians and infection control teams in hospitals as well as by physicians working in outpatient care. Infection and colonization were differentiated according to definitions from the Centers for Disease Control and Prevention (*7*) and interpreted by a physician.

Of the 1,098 primary MRSA isolates, 21 could not be digested by the restriction enzyme *Sma*I and were therefore investigated further (Table). All 21 patients (14 male, 7 female; median age 58 years, range 1–83 years) harbored MRSA of clonal lineage ST398. Although MRSA ST398 is suspected of being able to acquire virulence factor genes (*1*), only 5 patients were infected, whereas 15 were colonized. Status was unknown for 1. Of the 15 colonization cases, 12 were detected by screening. Regarding the infections, 4 cases were of minor clinical relevance, but 1 case (isolate no. 3332) showed progression of major clinical relevance: a 64-year-old pig farmer had received a prosthetic knee, and postoperative joint empyema with MRSA ST398 developed soon afterwards.

**Table:** Clinical MRSA isolates of clonal lineage ST398, Upper Austria, January 2006–May 2008*

Isolate	*spa* type	SCC*mec* group	Resistance	Patient age, y/ sex	Sample source	Diagnosis	Infected or colonized	Hospitalized	Profession, direct animal contact
2753	t2346	V	CLI, DOX, ERY	1/M	Throat	Pyodermatitis	I (SST)	Yes	Grandson of pig farmer (son of 2754), yes
2064	t011	V	CLI, DOX, ERY	54/M	Wound smear	Gouty tophus	I (SST)	Yes	Pig farmer, yes
3190	t011	V	CLI, DOX, ERY	48/M	Wound smear	Decubitus ulcers	I (SST)	Yes	Construction worker, no
3332	t011	V	DOX	64/M	Wound smear, joint puncture fluid	Joint empyema	I (BJ)	Yes	Pig farmer, yes
3509	t011	V	DOX	80/M	BAL	COPD	I (pneumonia)	Yes	Caster, unknown
1960	t011	V	DOX	62/M	Screening conjunctival swab	Paroxysmal tachycardia	C	Yes (ICU)	Farmer, yes
2023	t011	V	CLI, DOX, ERY	68/M	Screening wound smear	Peripheral arterial occlusive disease	C	Yes	Pig farmer, yes
2167	t011	V	DOX	47/M	Screening tracheal secretion	Epidural hematoma	C	Yes (ICU)	Agricultural worker, yes
2256	t011	V	DOX	73/M	Screening wound smear	Ulcus cruris	C	Yes	Cattle and pig farmer, yes
2726	t034	IV	–	50/M	Screening nose swab	Pneumothorax	C	Yes	Unknown
2754	t2346	V	CLI, DOX, ERY	33/F	Screening nose, throat swab	Premature delivery	C	No	Daughter of pig farmer, yes
2770	t011	V	CLI, DOX, ERY	81/F	Screening wound smear	Ulcus cruris	C	No	Farmer, yes
2832	t011	V	CLI, DOX, ERY	79/F	Screening wound smear	Ulcus cruris	C	No	Pig farmer, yes
2909	t011	V	DOX	79/M	Screening wound smear	Ulcus cruris	C	No	Pig farmer, yes
3078	t011	V	CLI, DOX, ERY	42/M	Screening nose swab	Erysipelas, septicemia	C	Yes	Farmer, yes
3195	t011	V	–	69/M	Wound smear	Intertrigo	C	Yes	Retired farmer (hens), yes
3335	t011	V	DOX	65/F	Screening axilla, nose, groin	Screening	C	No	Wife of pig farmer 3332, yes
3336	t011	V	DOX	32/M	Nose, wound smear	Screening	C	No	Son of pig farmers 3332 and 3335, yes
3391	t011	V	CLI, DOX, ERY	83/F	Feces	Polytrauma	C	Yes	Retired, no
3456	t011	V	DOX	69/F	BAL	Hemoptysis, bronchitis	C	Yes	Cleaner, no
3008	t011	V	DOX	48/F	Unknown	Unknown	Unknown	Unknown	Farmer, yes

*MRSA, methicillin-resistant *Staphylococcus aureus;* SCC, staphylococcal cassette chromosome; CLI, clindamycin; DOX, doxycycline; ERY, erythromycin; I, infected; SST, skin and soft tissue infection; C, colonized; BJ, bone and joint infection; BAL, bronchioalveolar lavage; COPD, chronic obstructive pulmonary disease; ICU, intensive care unit; –, no resistance found.

The first MRSA isolates in which ST398 was detected were collected in France during 1996–2002 (*8*). However, most publications concerning MRSA ST398 refer to samples collected since 2004 (*9*–*11*). In our institute, the first isolate belonging to ST398 was detected in January 2006, although we have investigated 2,657 MRSA isolates from persons all over Austria since 1996. Therefore, we assume that the emergence of MRSA ST398 in Austria is rather recent. Until now, this MRSA strain seemed to be restricted to Upper Austria, although pig farming is equally common in Lower Austria and in Styria. It might be interesting to investigate the reason for the varying prevalence of MRSA ST398 in different regions of Austria.

Our results correspond well with published data. SCC*mec* type V was predominant in our isolates, as has been found in Germany (*9*). One isolate harbored SCC*mec* type IV, but no type III, as has been reported in the Netherlands (*12*). None of our isolates harbored PVL genes, which confirms that the *lukF/lukS* genes are not necessarily present in community-acquired MRSA. Apparently, some community-acquired MRSA types (e.g., ST30, ST80, ST152) are PVL positive in most cases, whereas others (e.g., ST398) are not. However, although all the MRSA ST398 isolates found in Europe to date were PVL negative, a PVL-positive MRSA ST398 strain was recently detected in China (*13*).

The *spa* types among our isolates were t011, t034 (both commonly found in ST398), and t2346. They are closely related to each other as well as to other types belonging to ST398.

Antimicrobial drug–susceptibility testing showed that 19 of 21 isolates were resistant to doxycyline, 9 of which were also resistant to clindamycin and erythromycin. All isolates were susceptible to the other drugs tested. Only 2 isolates were fully susceptible to all agents tested. These antimicrobial drug–resistance profiles might reflect the frequent use of tetracyclines in veterinary medicine; in Austria, two thirds of all antimicrobial drugs used in veterinary practice, especially in pig and poultry farming, are tetracycline derivatives (*14*), a situation similar to that in other European countries (*11*,*12*). Therefore, it is not surprising that our resistance profiles correspond well with those from the Netherlands, Germany, and France (*9*,*12*,*15*).

The only isolate harboring *spa* type t034 was also the only isolate harboring SCC*mec* IV and is 1 of the 2 isolates that were fully susceptible to all antimicrobial drugs tested. Among all MRSA isolates, the percentage of ST398 in Upper Austria was 1.3% (6/463) in 2006, 2.3% (9/392) in 2007, and 2.5% (6/243) in 2008 (January–May). These percentages agree with data from Witte et al., who reported that MRSA ST398 is not frequent among *S. aureus* in Germany or the United Kingdom (*9*). However, the proportion of MRSA isolates that are ST398 has slightly increased in Upper Austria.

Most patients discussed in this article had had contact with animals. MRSA ST398 is known to be associated with animal contact, especially with pigs and cows (*8*–*10*,*12*). In the Netherlands and in France, the MRSA carriage rate is substantially higher for pig farmers and veterinarians than for the general population (*11*,*15*). In our study, 10 patients were pig farmers or direct relatives of pig farmers, and 6 were farmers (raised hens or unknown animal species). The animal contact status of 2 was unknown. In 2008, 3 of our patients had no direct animal contact; possible MRSA transmission from healthcare workers or other sources was not investigated. Thus, the question arises as to whether these isolates might represent more spread of this sequence type strain outside pig farms.

## Conclusions

MRSA of clonal lineage ST398 has emerged in humans in Austria. Moreover, it is not confined to Europe but has also been detected in China (*13*), Thailand, and Canada (*11*). This finding indicates a great potential for spread, quantitatively as well as geographically. Because the international meat and livestock market is active, the stage is set for rapid spread. In addition, the largest exporter of live pigs in Europe is the Netherlands, and up to 39% of pigs from the Netherlands carry MRSA in their nares (*12*). Thus, we suggest intensified establishment of collaborations between laboratories from different countries.
